# MiR-124-3p helps to protect against acute respiratory distress syndrome by targeting p65

**DOI:** 10.1042/BSR20192132

**Published:** 2020-05-27

**Authors:** Yufeng Liang, Junjie Xie, Di Che, Chunmin Zhang, Yongmin Lin, Lin Feng, Jinlu Chen, Jie Chen, Lihe Chen, Zhiyuan Wu

**Affiliations:** 1 Pediatric Intensive Care Unit, Guangzhou Women and Children’s Medical Center, Guangzhou Medical University, Guangzhou, Guangdong province, China; 2 Professor Wang Bin’s Famous Medicine Studio, Pediatric Department, Sanshui Women and Children’s Hospital, Foshan, Guangdong province, China; 3 Department of Clinical Biological Resource Bank, Guangzhou Institute of Pediatrics, Guangzhou Women and Children’ s Medical Center, Guangzhou Medical University, China; 4 Library, Guangzhou Women and Children's Medical Center, Guangzhou Medical University, Guangzhou, Guangdong province, China

**Keywords:** acute respiratory distress syndrome, apoptosis, inflammation, macrophage, miR-124-3p, p65

## Abstract

**Background:** Acute respiratory distress syndrome (ARDS) is a severe form of acute lung injury that has a high mortality rate and leads to substantial healthcare costs. MicroRNA-124-3p (miR-124-3p) helps to suppress inflammation during a pulmonary injury. However, its mechanism of action is largely unknown, and its role in ARDS remains to be determined.

**Methods:** Mice and NR8383 cells were exposed to lipopolysaccharides (LPS) to induce ARDS, and their miR-124-3p levels were determined. After a miRNA agomir was administrated to the mice, their pulmonary injuries were evaluated by H&E staining and assays for peripheral inflammatory cytokine levels. The direct interaction between miR-124-3p and p65 was predicted, and then confirmed by a luciferase activity assay. The role played by miRNA-124-3p in regulating p65 expression was further examined by transfection with its agomir, and its role in cell apoptosis was investigated by observing the effects of miRNA overexpression *in vitro* and *in vivo*.

**Results:** After exposure to LPS, there was a consistent decrease in miR-124-3p expression in the lungs of mice and in NR8383 cells. After treatment with the miR-124-3p agomir, the degrees of pulmonary injury (e.g. alveolar hemorrhage and interstitial edema), and the increases in IL-1β, IL-6, and TNF-α levels induced by LPS were significantly attenuated. Overexpression of miR-124-3p in NC8383 cells and lung tissues significantly suppressed LPS-induced p65 expression and cell apoptosis.

**Conclusions:** These results suggest that miR-124-3p directly targeted p65, and thereby decreased the levels of inflammation and pulmonary injury in a mouse model of ARDS.

## Introduction

Acute respiratory distress syndrome (ARDS) occurs when fluid builds up in the tiny elastic air sacs (alveoli) in lungs. The fluid prevents the lungs from filling with sufficient quantities of air, which results in less oxygen reaching the bloodstream. This deprives organs of the oxygen they need to properly function. Acute lung injury (ALI) is a widespread, variable type of lung injury characterized by a low oxygen level in the blood, non-cardiogenic pulmonary edema, low lung compliance, and widespread capillary leakage. The term acute lung injury was abandoned in the 2012 Berlin classification of acute respiratory distress syndrome, and this state is now referred to as mild ARDS. The clinical hallmarks of ARDS include hypoxemia and bilateral radiographic opacities, along with diffuse alveolar damage [1]. ARDS is histologically characterized by marked inflammatory cell infiltration, and causes severe damage to the lungs [2]. It has been estimated that 141,500 new cases of ARDS occur worldwide each year, with a yearly incidence of 58/100,000 individuals [3]. In ill patients, pulmonary inflammation is commonly present due to different etiologies, and often aggravates ARDS in patients being treated in ICUs. The incidence of ARDS in Japan was estimated to be 3.13 cases/100 hospital beds or 1.91 cases/ICU bed per year, and the most frequent underlying disease was pneumonia (34%), followed by sepsis (29%) [4]. ARDS is associated with a high rate of mortality, and 30–35% of patients with ARDS die as a result of the disease or its complications [3]. Although some therapies have been developed to treat ARDS, no therapies have shown the efficacy needed to reduce the mortality rate or prolong the life of ARDS patients.

It has been well accepted that ARDS is primarily induced by pathogenic inflammation, and macrophages are critical participants in that inflammation [5]. As essential components of the immune system, pulmonary macrophages represent the first line of host defense, as they recognize and scavenge airborne particles and microbes. Upon recognizing an endogenous or exogenous ligands, macrophages become activated via IRF- or NF-κB-dependent signaling pathways and promptly release inflammatory cytokines such as type I IFN, TNF-α, and IL-1β (Hiraiwa and van Eeden, 2013), leading to the initiation of inflammatory responses [6]. On the other hand, macrophages also help to resolve lung inflammation and repair tissue damage during the later phases of inflammatory disorders [7].

MicroRNAs (miRNAs) are a newly identified class of small RNA molecules with a length of 21–24 nucleotides, and primarily regulate protein expression at the post-transcriptional level [2]. They critically contribute to a multitude of biological pathways, and resultantly participate in various cellular processes such as apoptosis, proliferation (Bueno and Malumbres, 2011), and inflammatory responses (Hagen and Lai, 2008) [3]. Increasing numbers of studies have shown the important roles played miRNAs in pulmonary injuries, including acute lung injuries (ALI) and ARDS [3,8]. MiRNAs have also been suggested as potential biomarkers and therapeutic targets for treating ARDS [9]. A recent study revealed that a metformin-induced low level of miR-138-5p expression could cause an increase in SIRT1 expression that suppressed the MAPK pathway and alleviated ARDS [10]. MiRNA-124-3p has been reported to be expressed in various tissues, including lung, breast, brain, and gastrointestinal tissues. Aberrant expression of miRNA is highly associated with the initiation, development, or resolution of diseases, including cancers. Additionally, miRNAs have been shown to be associated with anti-inflammation activity in certain tissue injuries, including traumatic brain injuries and hepatic I/R injuries [11–13]. Furthermore, it has been shown that miR-124-3p can suppress inflammatory responses in traumatic acute lung injuries and thereby ameliorate those injuries [11]. A previous study of non-small cell lung cancers indicated that miR-124 helped to regulate autophagy, NF-kappa signaling, and cell viability, and that suppression of p62 expression by miR-124 was correlated with the NF-kappa subunit, RELA/p65 [14]. In a study of retinal microglia cells, it was shown that miR-124 directly controls Rac1 expression; furthermore, that study also revealed a miR-124-dependent mechanism in which Rac1 activation-mediated reactive oxygen species production stimulated p65 NF-kappa phosphorylation and induced the release of TNF-alpha from retinal microglial cells [15]. Additionally, cultures of normal rat NR8383 cells were established from normal rat alveolar macrophage (AM) cells obtained by lung lavage. It found that the NR8383 cells maintained their typical AM-like size, appearance, phagocytic behavior, and immunological properties over many passages. The applicability of the *in vitro* NR8383 AM assay suggests an alternative approach for regulatory hazard assessment. A previous study demonstrated that an ARDS model could be established by injecting LPS into mice that had previously received metformin. To further study the mechanism for that model, alveolar macrophages (NR8383 cells) were cultured *in vitro* and treated with LPS and metformin [10]; after which, phosphorylation levels were detected and regulatory relationships were analyzed [10]. In the present study, we observed the dysregulated expression of miR-124-3p in a mouse model of ARDS and also in NR8383 cells in order to investigate the mechanism by which miR-124-3p affects pulmonary injuries, and found that the mechanism might involve p65.

## Materials and methods

### Animals

Male Balb/c mice (8–10 weeks old, 22–26 g) were purchased from the Animal Center of Southern Medical University. The study protocol was approved by the Ethics Committee of Guangzhou Medical University. All animal experiments were conducted at the Experimental Animal Center of Guangzhou Medical University. After acclimatization for 5 days, the mice were randomly assigned to the following four groups consisting of eight mice per group: (1) Control group; (2) LPS alone (0.05 mg/mouse); (3) LPS+ negative control (NC); (4) LPS+miR-124-3p. After the mice were anesthetized by an i.p. injection of sodium pentobarbital (45 mg/kg), they underwent surgery to expose the trachea and right internal jugular vein. As previously described, the ARDS mouse models were established via a one-time i.t. instillation of 5 mg/kg LPS (Escherichia coli LPS serotype 0111: B4) given in 50 μl of sterile phosphate-buffered saline (PBS) with an 18-gauge catheter. The agomir (20 nmoles in 20 µl) of miR-124-3p (5′-UAAGGCACGCGGUGAAUGCCAA-3′) or the counterpart NC (5′-UUCUCCGAACGUGUCACGUTT-3′; GenePharma, Shanghai, China) was injected into the internal jugular vein of each mouse 3 h after an airway administration of LPS or vehicle (PBS). During the first 3 h between miRNA injection and killing, the mice were mechanically ventilated. Atracurium was infused intravenously to inhibit spontaneous respiration and maintain muscle relaxation. Mechanical ventilation was carried out with a small animal ventilator and using the following respiratory parameters: respiratory rate = 40 breaths/min, ventilation time = 4 h, inspiratory to expiratory time ratio = 1:2, oxygen concentration = 21%, and tidal volume = 30 ml/kg, based on previous studies [16,17]. No mouse died during the 24 h of observation following LPS administration. After 24 h, all animals were killed by carbon dioxide inhalation (airway administration of LPS or vehicle (PBS) → at 3 h post administration, agomir or the counterpart NC injection into the internal jugular vein → after injection, mechanical ventilation for 4 h → at 24 h post administration, mice were killed for study including histological observation). One lung of each mouse was collected and stored at −80°C and the other lung was collected and fixed in neutral formalin. The blood of each mouse was collected and then centrifuged to obtain the plasma, which was subsequently stored at −80°C until examination. All the mice were housed in SPF rooms that were maintained under controlled conditions of 20–26°C, 40–70% relative humidity, and a 12-h dark/light cycle. The mice had free access to food and water. All experimental procedures involving mice were performed according to instructions in the National Institutes of Health Guide for the Care and Use of Laboratory Animals, and the experimental protocols were approved by the Guangzhou Women and Children’s Medical Center affiliated with Guangzhou Medical University.

### Cell culture and treatment

NR8383 cells, a rat macrophage cell line, were obtained from the Cell Bank of the Chinese Academy of Sciences (Shanghai, China), and maintained in Ham’s F12 medium (Sigma-Aldrich, St. Louis, U.S.A.) containing 10% fetal bovine serum (FBS; Hyclone, Logan, UT, U.S.A.) in a humidified 37°C chamber containing 5% CO_2_. When the cells reached a density of 0.5 × 10^6^ cell/ml, they were seeded into six-well plates and cultured overnight. Next, Lipofectamine 2000 (Invitrogen, Carlsbad, CA, U.S.A.) was used to transfect the cells with 0.5 nmol of miR-124-3p agomir or NC under serum-free conditions. At ∼5 h after transfection, the medium was refreshed with new medium containing 10% FBS. After incubation for 24 h, the transfected cells were stimulated with 1 μg/ml of LPS for 12 h, and then harvested for examination.

### Luciferase activity assay

After target predictions were performed using the miRanda, starBase, and TargetScan databases, the wild type 3′UTR (WT 3′UTR) of the potential target containing the miR-124-3p binding sequence was cloned into a luciferase reporter plasmid (pMIR-Report; Ambion, Austin, TX, U.S.A.). Concurrently, the mutant binding site of 3′UTR (MUT 3′UTR) was inserted into the reporter plasmid and used as a NC. NR8383 cells previously transfected with miR-124-3p agomirs were transfected with the luciferase reported plasmid using Lipofectamine 2000. After ∼48 h of transfection, the cells were harvested and their luciferase activity was examined by the dual-luciferase activity assay system (Promega, Madison, WI, U.S.A.). The primers used were as follows:

P65 (WT) (forward): 5′-CCGCTCGAGAAATAACGCCCCAGATACCAGC-3′,
(reverse): 5′-ATTTGCGGCCGCACAACTTACCCTACTATTAAGGCACTTG-3′.
P65 (mut) (forward): 5′-CCGCTCGAGAAATAACGCCCCAGATACCAGC-3′,
(reverse): 5′-ATTTGCGGCCGCCCCCCACTCTTAACAACTTACCCTAC-3′.

### Real-time PCR

Trizol reagent (Invitrogen) was used to extract the total RNA from cultured cells and lung tissues according to the manufacturer’s protocols. Next, a 500-ng aliquot of purified RNA was reverse transcribed into cDNA by using a PrimeScript® miRNA cDNA Synthesis kit (Takara, Dalian, China). The cDNA was used to perform real-time (RT) PCR on an ABI 7500 PCR system (Applied Biosystems, Foster City, U.S.A.) with SYBR® Premix Ex Taq™ II (TaKaRa). The primers used were as follows:

miR-124-3p- forward:
  5′‐CTCAACTGGTGTCGTGGAGTCGGCAATTCAGTTGAGATCAAGGT‐3′,
  reverse: 5′‐ACACTCCAGCTGGGCGTGTTCACAGCGGAC‐3′;
miR-138-5p- forward:
  5′‐CTCAACTGGTGTCGTGGAGTCGGCAATTCAGTTGAGCGGCCTGAT‐3′,
  reverse: 5′‐ACACTCCAGCTGGGAGCTGGTGTTGTGAATCA‐3′;
U6- (forward): 5′‐CTCGCTTCGGCAGCACA‐3′,
reverse:5′‐AACGCTTCACGAATTTGCGT‐3′;
p65 –forward: 5′- CTTCCAAGAAGAGCAGCGTG-3′,
reverse: -5′- CCAGAGTTTCGGTTCACTCG -3′.

The reaction parameters were 2 min at 95°C, followed by a subsequent 40 cycles of 15 s at 95°C and 45 s at 60°C. Each sample was assayed in triplicate. The levels of miRNA expression were normalized to those for U6 snRNA, and calculated by the 2^−△△Ct^ method.

### Western blot assays

Samples of lung tissue and aliquots of cells were lysed in ice-cold cell lysis buffer (Beyotime Biotechnology, Haimen, China), and the total protein content in each sample was determined using a BCA Protein Assay Kit (Beyotime Biotechnology). Next, the protein samples were denatured at 95°C for 5 min; after which, they were loaded onto an 8–12% SDS-polyacrylamide gel and separated by electrophoresis. The separated protein bands were then transferred onto PVDF membranes (Millipore, Burlington, MA, U.S.A.), which were subsequently blocked with 5% nonfat milk at room temperature. The membranes were then incubated overnight at 4°C with primary antibodies against p65, Bax, Bcl-2, Caspase-3, and GAPDH (Santa Cruz Biotechnology, Dallas, TX, U.S.A.); after which, they were incubated with a horseradish peroxidase-conjugated secondary antibody at room temperature for 1 h. The immunostained protein bands were visualized by the enhanced chemiluminescence method.

### Enzyme-linked immuno sorbent assay (ELISA)

The collected mouse serum was thawed, and its levels of IL-1β, IL-6, and TNF-α were examined with ELISA Kits (R&D Systems Inc., Minneapolis, MN, U.S.A.). In brief, 0.1 ml of serum was added in duplicate to an ELISA plate coated with antibodies, and then incubated at room temperature for 1 h. After the addition of antibodies against IL-1β, IL-6 or TNF-α, and a subsequent 1 h incubation, streptavidin-HRP and 3,3′-5,5′ tetramethylbenzidin (TMB) were added. Finally, optical density was measured at 450 nm with a Multiskan Spectrum Microplate spectrometer (Thermo Fisher, Waltham, MA, U.S.A.), and the concentrations of IL-1β, IL-6, and TNF-α were calculated based on standard curves.

### Immunofluorescence

Expression of p65 was determined by immunostaining. After fixation with 4% PFA and permeabilization with 0.5% Triton X-100, the cultured cells were incubated for 1 h at room temperature with a primary antibody against p65 (Santa Cruz Biotechnology, Inc.). Subsequently, the cells were exposed to a fluorescein isothiocyanate-conjugated secondary antibody (Abcam, Cambridge, MA, U.S.A.). Finally, DAPI (Beyotime Biotechnology) was added to stain the nuclei. The immunostained cells were then observed at ×200 magnification under a Nikon Eclipse E1000 fluorescence microscope (Nikon Corporation, Tokyo, Japan), and representative images were recorded.

### Hematoxylin and Eosin (H&E) staining

Portions of lung tissue that had been fixed in neutral formalin were dehydrated by immersion in a series of ethanol solutions of increasing concentrations up to 100%. After being embedded with paraffin wax, the samples were cut into 5 μm-thick sections and then dewaxed. Next, the sections were rehydrated by immersion in a series of ethanol solutions of decreasing concentrations, and then stained with H&E. The stained sections were observed under a microscope for pathologic evaluation.

### Lung histology evaluation

Lung injury scores were calculated by evaluating the degrees of inflammatory cell infiltration, hemorrhage, interstitial and alveolar edema, and the thickness of the alveolar septum in five randomly selected fields in a blinded manner under a light microscope. This method was previously used by [18]. A score of 0 indicated no damage, l indicated mild damage, 2 indicated moderate damage, 3 indicated severe damage, and 4 indicated very severe histological damage.

### Apoptosis analysis

Apoptosis in cultured cells was determined by flow cytometry and Hoechst 33258 staining. For the flow cytometry assay, the harvested cells were fixed with pre-cooled 70% ethanol at 4°C overnight; after which, they were washed twice with PBS and then suspended in Annexin-V Binding Buffer at a density of 2–3 × 10^6^ cells/ml. Next, Annexin-V FITC and propidium iodide (PI) buffers were added to the cell suspension and the mixture was incubated at room temperature for 15 min while being protected from light. The cells were then analyzed by flow cytometry (Becton Dickinson, Franklin Lakes, NJ, U.S.A.). For the Hoechst 33358 (Sigma, U.S.A.) staining assay, the cultured cells were fixed with 4% PFA at room temperature for 15 min, and then incubated in the dark with Hoechst 33258 (10 μg/ml) solution for 5 min. The stained cells were then observed with a fluorescence microscope.

Cell apoptosis in samples of lung tissue was examined by the Terminal Transferase dUTP Nick End Labeling (TUNEL) assay. In brief, 5 μm-thick sections of lung tissue fixed in neutral formalin were prepared as described above and then incubated with a TUNEL reaction mixture (Beyotime, Shanghai, China) for 1 h at room temperature in a humidified incubator. TUNEL-positive cells, which displayed a brown color, were analyzed under a fluorescence microscope.

### Statistical analysis

All data were analyzed using IBM SPSS Statistics for Windows, Version 19.0 (IBM Corp, Armonk, NY, U.S.A.), and results are presented as the mean ± SD of data obtained from at least three independent experiments. Comparisons made between multiple groups were analyzed by using one-way analysis of variance (ANOVA) followed by the Tukey post-hoc test. The Student’s *t* test was used to analyze comparisons made between two groups. *P*-values < 0.05 were considered to be statistically significant.

## Results

### Decreased expression of miR-124-3p and miR-138-5p in the ARDS models

**Figure 1:**
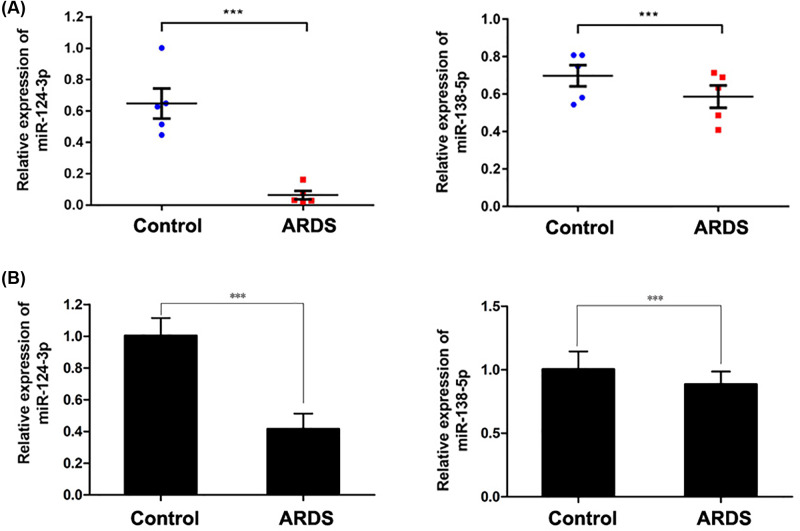
Down-regulated expression of miR-124-3p and miR-138-5p after exposure to LPS The levels of miR-124-3p and miR-138-5p expression in lung tissues of the ARDS model mice (**A**) and in NR8383 cells exposed to LPS (**B**) were determined; ***P<0.001 as compared with the control.

The levels of miR-124-3p and miR-138-5p expression were first determined in the mouse model of ARDS. As shown in Figure 1A, there were significant decreases in the levels of miR-124-3p and miR-138-5p expression in the mouse models. Subsequently, in Figure 1B, we examined the levels of miR-124-3p and miR-138-5p in NR8383 cells that had been exposed to LPS, and found that those levels were also significantly decreased when compared with the levels in control cells. These findings suggested that those miRNAs might assist in regulating the activity of macrophages, and thus be associated with ARDS. Consistent with our *in vivo* results, the decrease in miR-124-3p expression was more pronounced than the decrease in miR-138-5p expression, suggesting that miR-124-3p might be more involved in ARDS than is miR-138-5p. Therefore, miR-124-3p was selected for our further studies.

### Effect of miR-124-3p on ARDS

**Figure 2:**
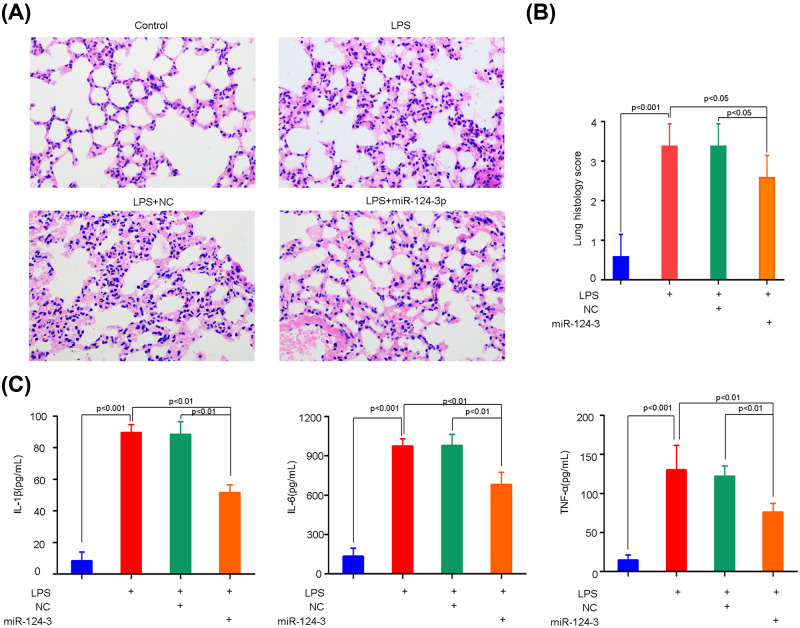
Effects of miR-124-3p on pulmonary injury and inflammation in ARDS mice The mice received miR-124-3p or the negative control (NC) at 3 h after LPS or control treatment, and were killed to collect samples of lung tissue and serum ∼24 h later. The pulmonary injuries were histologically evaluated by H&E staining (**A**). Lung histological alterations were assessed by a lung injury scoring system (**B**), and the inflammation responses were evaluated by determinations of serum IL-1β, IL-6, and TNF-α levels via ELISA (**C**).

To confirm the role of miR-124-3p in the pathogenesis of ARDS, the miRNA agomirs were given to the model mice via i.v. injection. After LPS treatment, severe diffuse pulmonary lesions were observed, including alveolar hemorrhage, interstitial edema, inflammatory cell infiltration, pulmonary congestion, and thickening of the alveolar wall (Figure 2A), indicating that the mouse ARDS model had been successfully established. However, the pathological changes induced by LPS were markedly alleviated by miR-124-3p treatment, indicating that miRNA might play a protective role in ARDS. Furthermore, all of the pathological changes in lung tissues became attenuated after administration of miR-124-3p, resulting in reduced lung injury scores (Figure 2B).

As inflammation contributes to ARDS, the inflammatory markers of ARDS in the lung tissues of the model mice were determined by ELISA. As expected, the pulmonary levels of IL-1β, IL-6, and TNF-α were markedly increased in the ARDS model mice (Figure 2C); however, those levels significantly decreased after the mice were treated with miR-124-3p (Figure 2C). These results indicated that miR-124-3p negatively regulates the levels of pro-inflammatory cytokines, and thereby reduces the inflammation associated with ARDS.

### MiR-124-3p directly targeted p65

**Figure 3:**
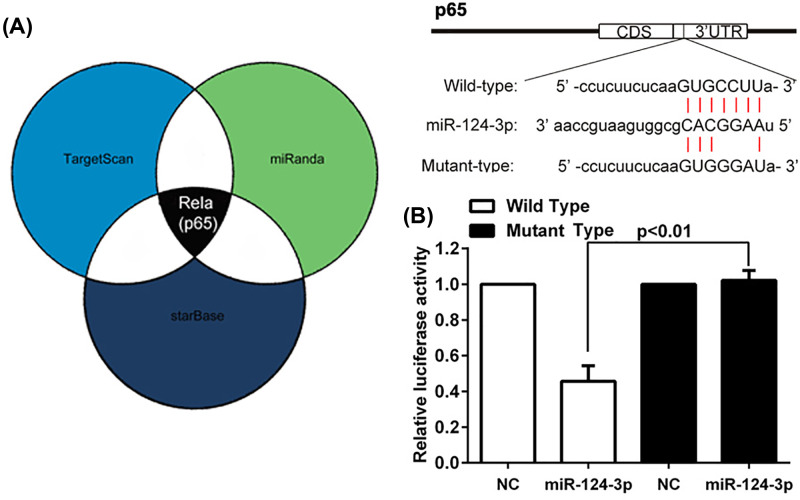
Identification of p65 as a direct target of miR124-3p p65 mRNA was predicted to be a direct target of miR-124-3p by the TargetScan, miRanda, and starBase databases (**A**), and direct interaction was demonstrated by a luciferase report assay (**B**).

To investigate the possible mechanism by which miR-124-3p regulates the inflammatory responses associated with ARDS, the target for miR-124-3p was predicted by examining various databases, and then verified by a luciferase report assay. As shown in Figure 3A, the cross prediction made by using the TargetScan, miRanda, and starBase databases indicated that p65 mRNA was a direct target of miR-124-3p. As expected, miR-124-3p transfection significantly decreased the luciferase activity in cells with WT’ UTR transfection, but not with MT 3′ UTR transfection (Figure 3B).

**Figure 4:**
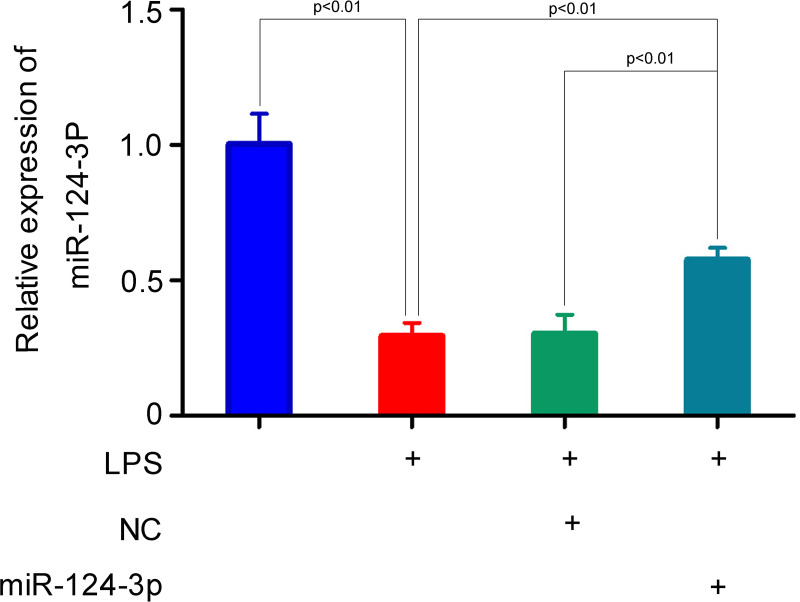
Mir-124-3p was transfected into NR8383 cells, and then detected by RT-PCR after LPS treatment

**Figure 5:**
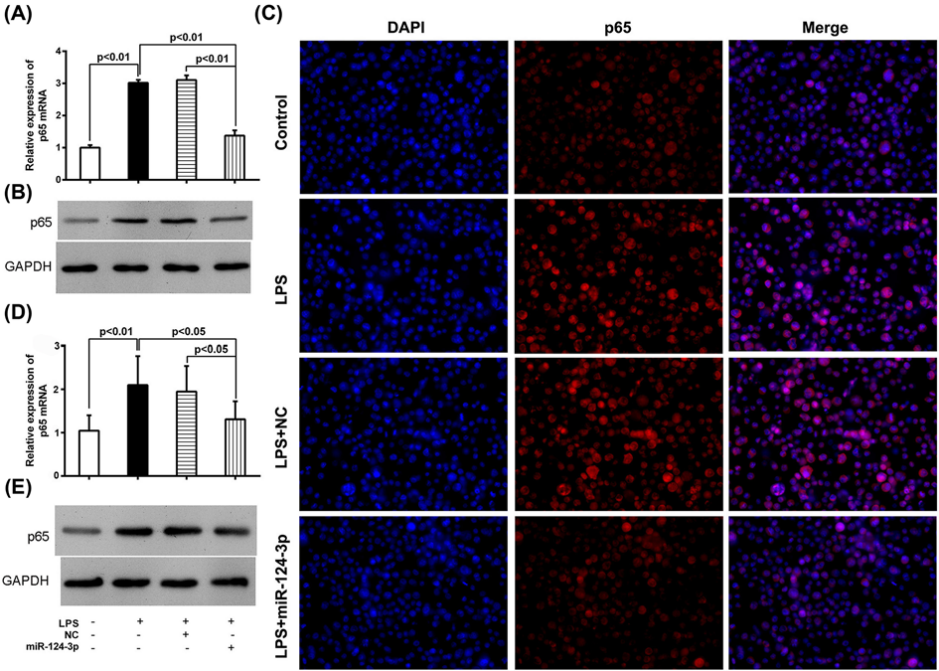
Effects of miR-124-3p on p65 expression The parent NC- or miR-124-3p-transfected NR8383 cells received control or LPS treatment for 12 h, and were then examined for miR-124-p expression by RT-PCR (**A**), Western blotting (**B**), and immunofluorescence (**C**). Approximately 3 h after LPS or control treatment, the mice were given miR-124-3p or the negative control (NC), and 12 h later, were examined for miR-124-3p expression by RT-PCR (**D**) and Western blotting (**E**).

Subsequently, the targeting of p65 mRNA by miR-124-3p was further confirmed in NR8383 cells and in mice. First, we transfected miR-124-3p into NR8383 cells, which led to significantly increased levels of miRNA-124-3p in cells exposed to LPS (Figure 4). This indicated that the miRNA had been successfully transfected into the cells, and they were appropriate for further use. In the parent NR8383 cells, p65 expression at the mRNA and protein levels was significantly increased in the LPS treatment group as compared with the control group (Figure 5A,B). As expected, the levels of p65 after LPS treatment were significantly lower in the miR-124-3p transfected cells than in the NC transfected cells. Additional immunofluorescence staining of the cells exposed to LPS showed a consistent decrease in immunostaining density after miR-124-3p transfection, as compared with immunostaining in the NC transfected cells (Figure 5C). Furthermore, similar changes in p65 expression were noted in samples of whole lung tissue from the *in vivo* experiments (Figure 5D,E). All these results further confirmed that p65 could be regulated by miR-124-3p both *in vitro* and *in vivo*.

### Effect of miR-124-3p on cell apoptosis

**Figure 6:**
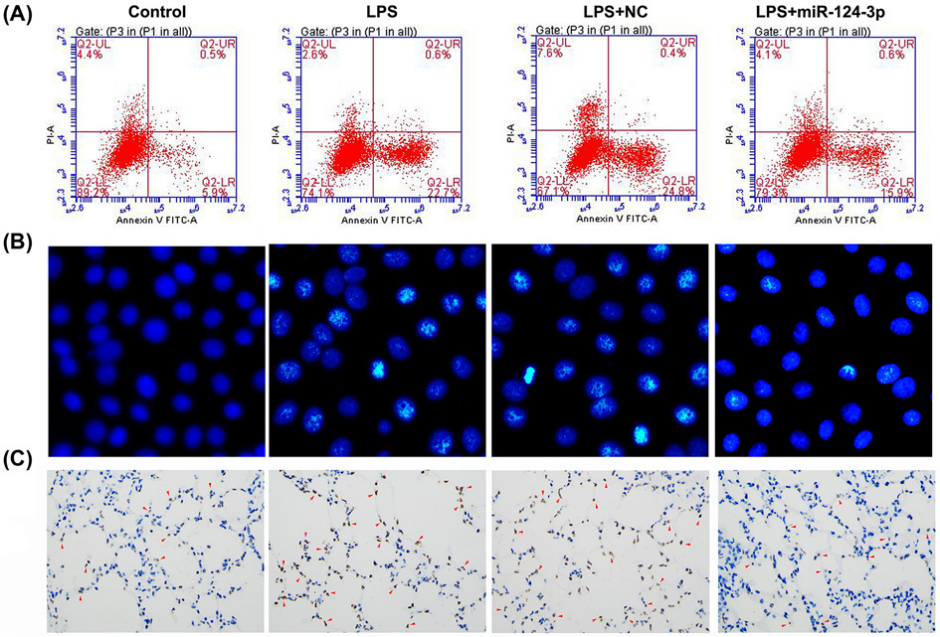
Effects of miR-124-3p on cell apoptosis induced by LPS treatment The parent NR8383 cells or cells transfected with miR-124-3p or the NC received control or LPS treatment for 12 h. Cell apoptosis was determined by flow cytometry (**A**), Hoechst staining (**B**), and the TUNEL assay (**C**). The red arrowhead indicates a TUNEL-positive cell.

The role of miR-124-3p in macrophage apoptosis was investigated both *in vitro* and *in vivo*. A flow cytometry analysis of NR8383 cells showed that the percentage of apoptotic cells was markedly higher in the LPS treatment group when compared with the control group, indicating that LPS could significantly induce macrophage apoptosis *in vitro* (Figure 6A). However, the percentage of apoptotic cells among LPS-treated cells was significantly decreased after miR-124-3p transfection when compared with the percentage of apoptotic cells among cells transfected with the NC. This decrease in apoptosis was also shown by Hoechst staining and the TUNEL assay (Figure 6B,C). These data indicated that the effect of LPS on cell apoptosis was significantly suppressed by miR-124-3p.

## Discussion

The treatment of ARDS remains a major challenge in clinics throughout the world, due to its high rates of morbidity and mortality. It is well accepted that NF-κB is closely associated with ARDS because of the important role it plays in managing inflammation; however, the mechanism by which NF-κB activity is regulated remains largely unknown. In the present study, we provided evidence, for the first time, that miR-124-3p can directly target p65, an essential subunit of NF-κB, and thereby inhibit the inflammatory response in lung tissue. Furthermore, we showed that forced overexpression of miR-124-3p helped to protect against ARDS.

ARDS is always characterized and caused by inflammatory responses that are accompanied by elevated levels of pro-inflammatory cytokines. In the present study, elevated levels of the pro-inflammatory cytokines IL-1β, IL-6, and TNF-α were observed in ARDS mice, and this was consistent was previous studies [19,20]. In addition to ALI/ARDs, cytokines, as typical pro-inflammatory factors, contribute to tissue injuries, such as acute liver and kidney injuries. It has been observed that cytokines not only regulate cell apoptosis, but also cause inflammation by triggering an intense infiltration of neutrophils and mediating the detrimental role of regulatory T cells [21,22].

The NF-κB signaling pathway regulates the expression of numerous inflammatory cytokines, including IL-1β, IL-6, and TNF-α [23]. Moreover, it’s increasingly acknowledged that excessive activation of NF-κB fundamentally contributes to the pathogenesis of ARDS; possibly by inducing the expression of pro-inflammatory mediators [24,25]. Consistent with that idea, it was observed that decreased NF-κB activity, as indicated by decreased p65 levels, occurs in conjunction with decreased plasma levels of IL-1β, IL-6, and TNF-α, and the attenuation of lung tissue injuries. Recently, several studies have shown the protective effect of attenuated NF-κB activity on ALIs. Cao et al. pointed out that Ulinastatin, a serine protease inhibitor, ameliorates LPS-induced acute lung injuries by suppressing TLR4/NF-κB signaling [26]. Furthermore, Hussain showed that inhibition of NF-κB by CT-133, a CRTH33 antagonist, also relieves LPS-induced lung injuries [21].

MiR-124-3p has been reported to play a variety of roles in many physiological and pathological processes. Increasing numbers of studies have shown that miR-124-3p functions as a suppressor molecule in many types of human cancers, including breast cancer, pancreatic ductal adenocarcinoma, hepatocellular carcinoma, and bladder cancer [27–30]. This effect was demonstrated by the ability of miR-124-3p to inhibit cancer cell proliferation and metastasis, and promote cell apoptosis. Additionally, growing evidence suggests an important regulatory role for miR-124-3p in inflammation. Shan et al*.* recently reported that increased miR-124-3p expression suppressed neuronal inflammation, and might serve as a biomarker for brain ischemic stroke [12,13,31]. In a case of hepatic ischemia/reperfusion injury, miR-124-3p inhibited inflammation and thereby improved the hepatic injury [13]. Moreover, miR-124-3p also demonstrated anti-inflammatory effects, and decreased the expression of inflammatory cytokines (e.g., IL-1β, IL-6, and TNF-α) in spinal microglial cells that had been incubated with LPS [32]. Consistent with those findings, we found that mrR-124-3p could inhibit inflammation in our present study.

The pathogenesis of ARDS has been ascribed to a variety of cells including macrophages, regulatory T cells, and epithelial cells. As critical secretors of IL-1β, IL-6, and TNF-α, macrophages play an important role in the initiation, development, and resolution of ARDS. Upon stimulation in ALI/ARDS, pulmonary macrophages are immediately activated in response to an infection-induced activation of toll-like receptors (TLRs) or other recognition receptors, leading to NF-κB activation and the production of pro-inflammatory cytokines [33–35]. Based on their response to stimulation, macrophages exist in one of two polarized states: a classically activated phenotype (M1) and an alternatively activated phenotype (M2) [36]. M1 macrophages exert pro-inflammatory effects, while M2 macrophages exert anti-inflammatory effects. Furthermore, it has been demonstrated that M1 macrophages play a critical role in the pathogenesis of ALI via their pro-apoptotic and pro-inflammatory effects [37]. A recent study showed that miR-124-3p was highly overexpressed in M1 macrophages as compared with M2 macrophages [38], indicating that miR-124-3p might promote the M2 polarization of macrophages. Additionally, the data in the present study showed that miR-124-3p directly targets NF-κB mRNA, which leads to an accumulation of M1 macrophages and their subsequent production of pro-inflammatory cytokines [35]. Therefore, we hypothesized that miR-124-3p might shift macrophages into the M2 subtype by decreasing NF-κB activity, and this possibility will be investigated in subsequent studies in our laboratory. Our preliminary data indicated that the typical histologic findings of ARDS were attenuated by miR-124-3p through its ability to directly target p65 and suppress NF-κB activity. However, a limitation of the present study is that no effect on gas exchange was investigated; although no mouse died during the 24-h observation period following LPS injection. In addition, the therapeutic effect and safety of miR-124-3p will require further extensive evaluation based on experience gained in clinical practice.

In conclusion, our study revealed that p65 mRNA is a direct target of miR-124-3p, and such targeting inhibited the production of pro-inflammatory cytokines and promoted the apoptosis of macrophages. Furthermore, miR-124-3p was shown to help protect against ARDS, possibly due to its ability to inhibit inflammation. These findings suggest miRNA-124-3p as a promising therapeutic target for treating ARDS.

## Abbreviations

ALI: acute lung injuries
ARDS: acute respiratory distress syndrome
H&E: Hematoxylin and Eosin
LPS: lipopolysaccharides
PI: propidium iodide
TLR: toll-like receptor
TUNEL: Terminal Transferase dUTP Nick End Labeling
