# Expression of Concern: MiR-124-3p helps to protect against acute respiratory distress syndrome by targeting p65

**DOI:** 10.1042/BSR20192132_EOC

**Published:** 2026-08-07

**Authors:** Yufeng Liang, Junjie Xie, Di Che, Chunmin Zhang, Yongmin Lin, Lin Feng, Jinlu Chen, Jie Chen, Lihe Chen, Zhiyuan Wu

**Affiliations:** 1 Pediatric Intensive Care Unit, Guangzhou Women and Children's Medical Center, Guangzhou Medical University, Guangzhou, Guangdong province, China; 2 Professor Wang Bin's Famous Medicine Studio, Pediatric Department, Sanshui Women and Children's Hospital, Foshan, Guangdong province, China; 3 Department of Clinical Biological Resource Bank, Guangzhou Institute of Pediatrics, Guangzhou Women and Children' s Medical Center, Guangzhou Medical University, China; 4 Library, Guangzhou Women and Children's Medical Center, Guangzhou Medical University, Guangzhou, Guangdong province, China

**Keywords:** acute respiratory distress syndrome, apoptosis, inflammation, macrophage, miR-124-3p, p65

*Bioscience Reports* has been made aware of potential issues surrounding the scientific validity of this paper and is hence issuing a permanent Expression of Concern to notify readers. Concerns have arisen regarding the peer-review process of this article, and a duplication was identified between images submitted to the Journal in the original version of the manuscript, which were later replaced. These duplications specifically involved:

- High similarity between the Figure 2A second panel (original, replaced image) and the Figure 1A LPS panel of Wei et al. 2020 (DOI: 10.1038/s41419-020-02857-4),
- High similarity between the Figure 2A third panel (original, replaced image) and the Figure 1A LPS+HFL-1 also from Wei et al. 2020.

The authors provided raw data for the histology images that included the published versions of these panels, but the originally submitted images were not present in the data provided. Original scans of the western blots were also provided but with a labelling discrepancy.

As such, the article will remain published but with an associated Expression of Concern.

